# Crimean-Congo Hemorrhagic Fever Virus Entry into Host Cells Occurs through the Multivesicular Body and Requires ESCRT Regulators

**DOI:** 10.1371/journal.ppat.1004390

**Published:** 2014-09-18

**Authors:** Olena Shtanko, Raisa A. Nikitina, Cengiz Z. Altuntas, Alexander A. Chepurnov, Robert A. Davey

**Affiliations:** 1 Department of Virology and Immunology, Texas Biomedical Research Institute, San Antonio, Texas, United States of America; 2 Laboratory of Regulation of Immunopoiesis, Institute for Clinical Immunology, Novosibirsk, Russian Federation; 3 Texas Institute of Biotechnology Education and Research, North American University, Houston, Texas, United States of America; Division of Clinical Research, United States of America

## Abstract

Crimean-Congo hemorrhagic fever virus (CCHFV) is a tick-borne bunyavirus causing outbreaks of severe disease in humans, with a fatality rate approaching 30%. There are no widely accepted therapeutics available to prevent or treat the disease. CCHFV enters host cells through clathrin-mediated endocytosis and is subsequently transported to an acidified compartment where the fusion of virus envelope with cellular membranes takes place. To better understand the uptake pathway, we sought to identify host factors controlling CCHFV transport through the cell. We demonstrate that after passing through early endosomes in a Rab5-dependent manner, CCHFV is delivered to multivesicular bodies (MVBs). Virus particles localized to MVBs approximately 1 hour after infection and affected the distribution of the organelle within cells. Interestingly, blocking Rab7 activity had no effect on association of the virus with MVBs. Productive virus infection depended on phosphatidylinositol 3-kinase (PI3K) activity, which meditates the formation of functional MVBs. Silencing Tsg101, Vps24, Vps4B, or Alix/Aip1, components of the endosomal sorting complex required for transport (ESCRT) pathway controlling MVB biogenesis, inhibited infection of wild-type virus as well as a novel pseudotyped vesicular stomatitis virus (VSV) bearing CCHFV glycoprotein, supporting a role for the MVB pathway in CCHFV entry. We further demonstrate that blocking transport out of MVBs still allowed virus entry while preventing vesicular acidification, required for membrane fusion, trapped virions in the MVBs. These findings suggest that MVBs are necessary for infection and are the sites of virus-endosome membrane fusion.

## Author Summary

Crimean-Congo hemorrhagic fever virus (CCHFV) is the cause of a severe, often fatal disease in humans. While it has been demonstrated that CCHFV cell entry depends on clathrin-mediated endocytosis, low pH, and early endosomes, the identity of the endosomes where virus penetrates into cell cytoplasm to initiate genome replication is unknown. Here, we showed that CCHFV was transported through early endosomes to multivesicular bodies (MVBs). We also showed that MVBs were likely the last organelle virus encountered before escaping into the cytoplasm. Our work has identified new cellular factors essential for CCHFV entry and potential novel targets for therapeutic intervention against this pathogen.

## Introduction

Crimean-Congo hemorrhagic fever virus (CCHFV) is a tick-borne virus causing outbreaks of severe hemorrhagic disease in humans, with a fatality rate approaching 30%. The virus is endemic to much of Eastern Europe, the Middle East, Asia, and Africa, although recent studies have detected CCHFV in ticks collected in Spain, indicating an expanding geographic distribution [1]–[4]. Despite the high mortality and global distribution of CCHFV, there are presently no licensed therapeutics to prevent or treat the disease.

CCHFV belongs to the family *Bunyaviridae*. It is an enveloped, pleomorphic virus, possessing a tripartite single-stranded RNA genome in negative orientation. The small segment, S, encodes the nucleocapsid protein N, whose role is to encapsidate viral RNA during transcription and genome replication. The large segment, L, encodes the RNA-dependent RNA polymerase, which associates with N to form the viral polymerase complex [5]. The medium segment, M, contains the gene for the viral glycoprotein polyprotein, which is co-translationally cleaved and post-translationally modified to generate two structural transmembrane proteins, Gc and Gn, and non-structural proteins GP38 and NSm [5]–[9]. The Gc and Gn form complexes on the virion surface and are responsible for binding to the cellular receptors and subsequent fusion of the viral envelope with host membranes [10], [11].

Virus entry into the cell is the first and critical step in the virus replication cycle. The host receptor of CCHFV has not been identified, although a recent study has suggested that nucleolin plays a necessary role in virus entry and that Gc is essential for binding to the cell [12]. Virus uptake occurs through clathrin-dependent endocytosis and requires cholesterol and low pH to productively infect host cells [13], [14]. An earlier study implicated Rab5, a small GTPase critical for vesicular transport from the plasma membrane to early endosomes, as important for infection [13]. The lack of the involvement of Rab7, which controls vesicular trafficking to late endosomes, in infection and the requirement for pH 6.0 to inactivate the virus led to speculation that virus fusion with host membranes takes place at early endosomes [13], although direct localization of virus to the early endosomes was not demonstrated. Little else is known about trafficking of the virus along the endocytic route or the identity of cellular compartments where the fusion of the viral and host membranes takes place. Here, we investigated the mechanism of transport of CCHFV through the host cell in more detail.

## Results

### CCHFV traffics through early endosomes in a Rab5-dependent manner

**Figure 1:**
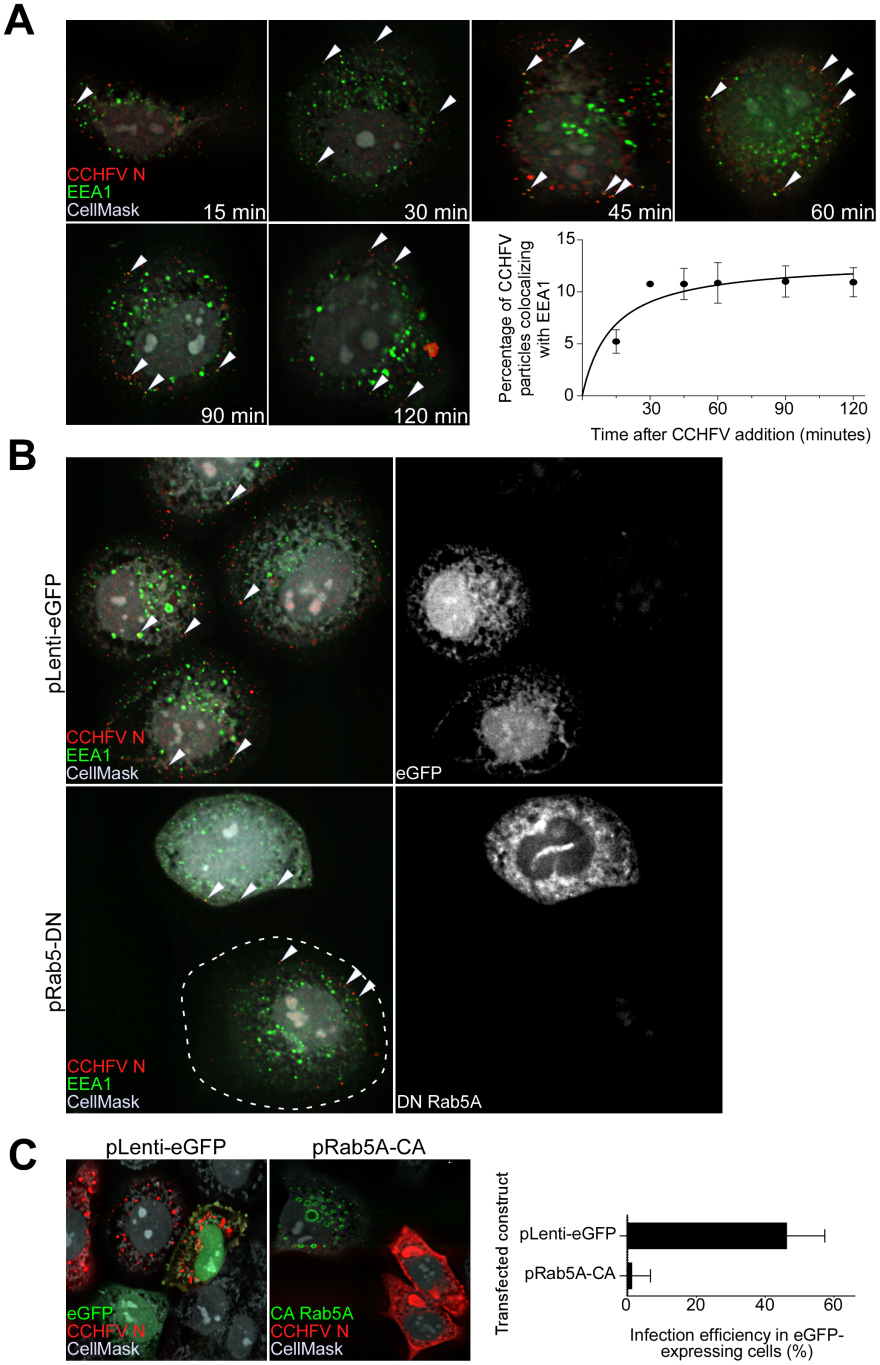
CCHFV traffics through early endosomes in a Rab5-dependent manner. (A) SW13 cells were incubated with CCHFV for indicated times. Then, the cells were fixed, permeabilized, and treated with CellMask blue dye (grey) to stain the cytoplasm and nucleus, anti-N antibody (virus, red), and anti-EEA1 antibody (early endosomes, green). Images were obtained as Z-stacks, and three-dimensional (3D) images of cells were generated to assess colocalization between virus and endosomes. The percentages of virus particles localizing to early endosomes were counted in 20 cells in each sample, and averages and standard deviations are shown. Arrowheads point to examples of CCHFV N-EEA1 colocalization (yellow). (B) SW13 cells were transfected with either pLenti-eGFP or pRab5A-DN. After 24 h, cells were incubated with CCHFV for 30 min, fixed, and stained with anti-N antibody (red), anti-EEA1 antibody (green), and CellMask blue dye (grey). eGFP-expressing cells are pseudocolored white (right panels). Images were generated and analyzed as described in (A). The edge of the cell at lower left is indicated by a dashed line. (C) SW13 cells transfected with either pLenti-eGFP or pRab5A-CA were incubated with CCHFV for 24 h. Subsequently, samples were fixed and treated with anti-N antibody (red) to detect infected cells and CellMask blue dye (grey) to define cell boundaries. Samples were imaged as described above. The number of eGFP-expressing cells that became infected (expressing N) is shown at right. The results are averages of three independent experiments, and the bars represent standard deviations.

CCHFV enters cells by clathrin-mediated endocytosis [13], [14]. The virus was also previously shown to require Rab5 during infection of cells [13], suggesting a requirement for trafficking through early endosomes. However, localization of the virus to early endosomes has not been demonstrated, and so we examined uptake and trafficking kinetics of virus particles in more detail. Human adenocarcinoma cell line, SW13, has been demonstrated to support CCHFV replication [15] and, therefore, was utilized in our study. SW13 cells were incubated with CCHFV for up to 120 min and then stained with antibodies to virus nucleoprotein N and early endosome antigen 1 (EEA1), which localizes exclusively to early endosomes [16]. CCHFV is known to enter cells within 90 min [14], and therefore the N protein signal was likely to represent incoming virions. We observed that virus localized to early endosomes as early as 15 min and reached steady state by 30 min, with approximately 10% of the virions being EEA1-associated (Fig. 1A). Our results directly confirm that CCHFV is trafficked through early endosomes during infection.

To test whether Rab5A facilitates CCHFV movement to early endosomes, we expressed a fusion of enhanced green fluorescent protein (eGFP) and the dominant negative form of the Rab5A containing a substitution of serine to asparagine at position 34 (DN Rab5A) in SW13 cells and then infected the cells with wild-type virus. The control included cells expressing eGFP. The DN Rab5A arrests transport of vesicles from the cell surface due to a block in maturation of early endosomes [17], [18] and, therefore, we expected to observe accumulation of virions near the cell surface. Indeed, cells expressing the DN, but not eGFP, showed virions localized close to the plasma membrane, in EEA1-positive structures (Fig. 1B). This finding confirms that CCHFV is transported in a Rab5A-dependent manner.

While expression of DN Rab5A inhibits formation of early endosomes, expression of a constitutively active mutant of Rab5A containing a substitution of glutamine to leucine at position 79 (CA Rab5A) enhances the formation of the endosomes, making the vesicles appear drastically enlarged, but also compromises maturation of and cargo transport to later endosomal compartments [19]–[22]. To determine whether CCHFV entry involves passage through endosomal compartments downstream of early endosomes, SW13 cells expressing a fusion of eGFP and CA Rab5A or eGFP alone as a control were infected with CCHFV for 24 h to allow virus to establish replication sites [13], [14]. The N distribution in eGFP-expressing cells was similar to that seen in untransfected cells and likely represented pools of newly expressed protein due to virus gene expression and genome replication. In contrast, in CA-expressing cells, N staining was limited and likely represented incoming virus trapped in endosomes, consistent with that described in the above figures (Fig. 1C, left and middle panels). The percentages of eGFP-expressing cells that were infected with CCHFV were found to be 47% and 1% in control eGFP- and CA-expressing cells, respectively (Fig. 1C, right panel). These data demonstrate that productive infection by CCHFV requires trafficking through early endosomes as well as downstream endosomal compartments.

### CCHFV localizes to MVBs during infection

**Figure 2:**
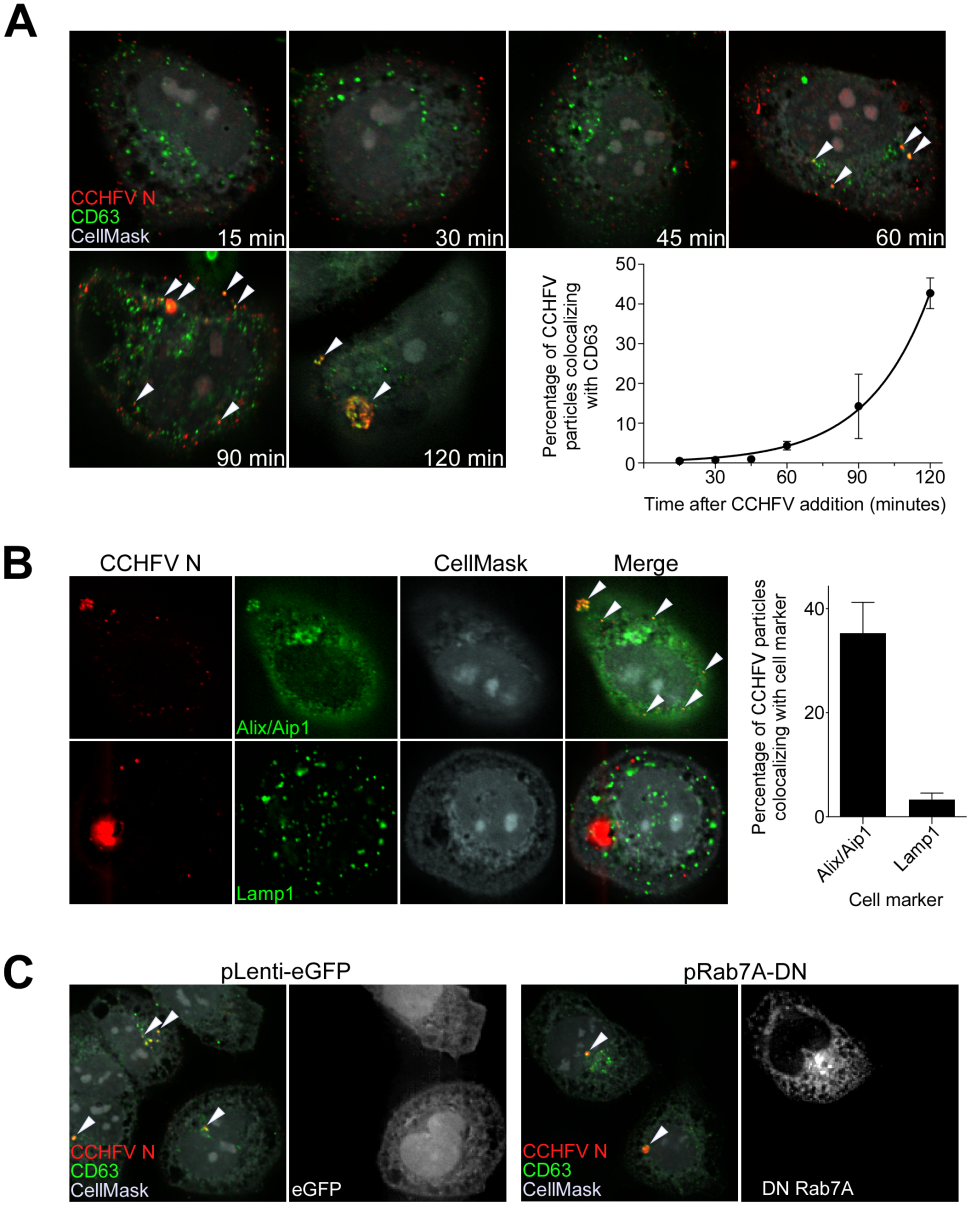
CCHFV localizes to and redistributes MVBs during infection. (A) SW13 cells were incubated with CCHFV for indicated times. Subsequently, the samples were fixed, permeabilized, and stained with anti-N antibody (red), anti-CD63 antibody (MVBs, green), and CellMask blue dye (grey). Images were generated and analyzed as described in Figure 1A. Arrowheads point to examples of CCHFV N-CD63 colocalization (yellow). (B) SW13 cells were incubated with CCHFV for 2 h, then fixed and treated with anti-N antibody (red) and either anti-Alix/Aip1 (green; upper row) or anti-Lamp1 (green; lower row) antibody. To define cell boundaries, samples were stained with CellMask blue dye (grey). Images were obtained and analyzed as described in Figure 1A. Examples of N-Alix/Aip1 colocalization (yellow) are indicated with arrowheads. Colocalization was quantified by counting the number of N puncta overlapping with Alix/Aip1 or Lamp1 staining (right panel). (C) SW13 cells were transfected with either pLenti-eGFP or pRab7A-DN. Twenty-four h later, cells were incubated with CCHFV for 120 min, then fixed and stained with anti-N antibody (red), anti-CD63 antibody (green), and CellMask blue dye (grey). eGFP-expressing cells are pseudocolored white (right panel of each pair). Images were generated and analyzed as described in Figure 1A.

Over time, cellular cargo from early endosomes can become concentrated on intraluminal vesicles within vacuolar domains, generating multivesicular bodies (MVBs) [23], [24]. Several viruses traffic through the MVB and depend on its sorting machinery during early steps in infection, including influenza A virus, vesicular stomatitis virus (VSV), Lassa fever virus (LASV), and lymphocytic choriomeningitis virus (LCMV) [25]–[28]. To test whether CCHFV localizes to MVBs during entry, we incubated SW13 cells with virus for up to 120 min and then stained them for N and tetraspanin protein CD63, which is enriched in MVBs [29]. As shown in Fig. 2A, virus localized to CD63-positive structures, presumably MVBs, starting at 60 min post-infection. This association appeared to accelerate over time until a major redistribution of the organelle occurred within the cell (Fig. 2A). CD63 is also known to contain a putative lysosomal targeting signal and to colocalize with late endosomal/lysosomal-associated membrane proteins Lamp1 and Lamp2 [30], suggesting that a fraction of this protein is in late endosomes and lysosomes. To address the question whether CCHFV localizes to these endosomal compartments during infection, we infected cells with CCHFV for 120 min and then stained them with antibodies detecting either *(i)* N and ALG-2-interacting protein X/apoptosis-linked-gene-2-interacting protein 1 (Alix/Aip1), which associates with MVBs to coordinate vesicle formation and biogenesis [31]; or *(ii)* N and Lamp1. As shown in Fig. 2B, 35% of CCHFV particles localized with Alix/Aip1, while only 3% of virions were found in Lamp1-positive endosomes. While it is possible that the Lamp1-positive endosomes represent late endosomes or lysosomes, the relevance of the association to virus infection mechanism is questionable since Rab7, which controls vesicular transport out of MVBs [32], does not play a role in CCHFV infection [13]. Thus, our findings demonstrate that virus is transported through MVBs during early stages of infection.

Several studies have reported that Rab7 controls cargo movement out of early endosomes [33], [34], while others indicate the function of this Rab later in the endocytic pathway, from MVBs to lysosomes [32]. To test whether Rab7A has a role in virus transport to MVBs in SW13 cells, we overexpressed the DN form of Rab7A, containing a substitution of threonine to asparagine at position 22 [35], in cells, challenged them with virus, and then tested for localization of virus to CD63-positive compartments. As a control, we transfected cells with a plasmid expressing eGFP alone. The overexpression of the DN or eGFP had no effect on localization of the virus to the MVBs (Fig. 2C), suggesting that Rab7A does not control virus transport between early endosomes and MVBs. Because Rab7 was shown as not important for CCHFV infection [13], our findings also indicate that CCHFV might not traffic beyond MVBs.

### CCHFV infection requires phosphatidylinositol 3-kinase (PI3K) activity

**Figure 3:**
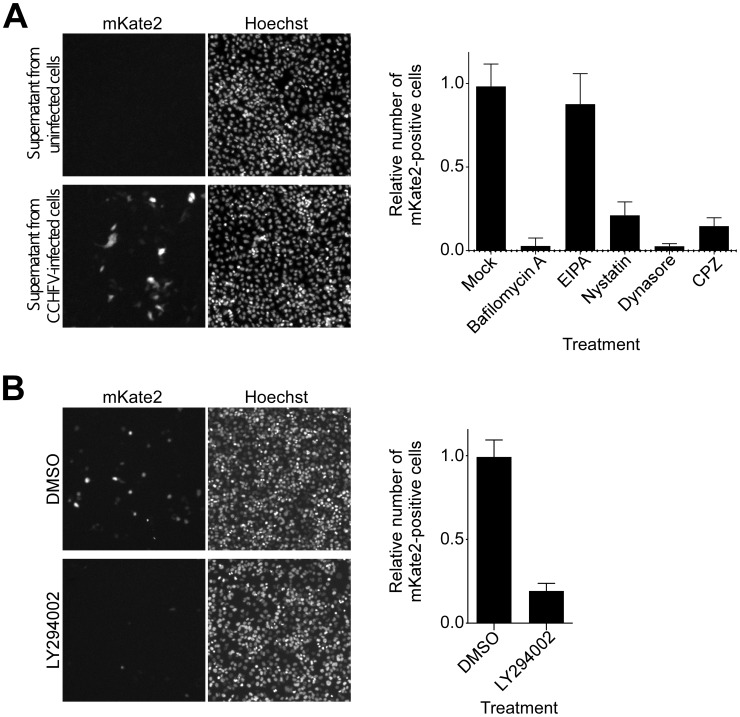
CCHFV infection depends on PI3K activity. (A) To generate CCHFV-mKate2, BsrT7/5 cells transfected with pT7-mKate2, pcDNA-N, and pcDNA-L were either mock-infected or infected with CCHFV. After 48 h, supernatants were transferred onto SW13 cells for 24 h. Cells were subsequently fixed, stained with Hoechst 33342 dye to identify nuclei, and photographed (left and middle panels). To assess the effect of pharmacological inhibitors on CCHFV-mKate2 infection, SW13 cells were pretreated with one of the following: bafilomycin A (20 nM), EIPA (10 µM), nystatin (100 µM), dynasore (200 µM), or CPZ (10 µg/mL). After 1 h, cells were incubated with the virus in the presence of the drug for 24 h. Subsequently, cells were fixed, stained with the Hoechst 33342 dye, and imaged. Numbers of nuclei and mKate2-positive (infected) cells were counted using CellProfiler software. The relative infection efficiencies were calculated by dividing the number of infected cells by the number of nuclei. The infection efficiencies are averages of three independent experiments, and standard deviations are shown (right panel). (B) SW13 cells were preincubated with DMSO or LY294002 (75 µM) for 1 h and then challenged with CCHFV-mKate2 in the presence of the drug. Twenty-four h later, cells were fixed, stained, and analyzed as in (A).

According to our data, CCHFV colocalizes with and redistributes MVBs during early stages of infection. It is unclear, however, whether there is a functional significance of this association. To study CCHFV infection in more detail, we generated CCHFV that incorporated a minigenome segment encoding the red fluorescent protein mKate2 (CCHFV-mKate2). BsrT7/5 cells [36] were transfected with the minigenome construct and plasmids expressing viral N and polymerase and then mock-infected or infected with wild-type CCHFV. The recombinant virus was collected 48 h after infection and inoculated onto fresh SW13 cells to assess packaging of the minigenome. As seen in Fig. 3A, inoculation of the supernatant from the cells infected with the virus resulted in the expression of the mKate2 gene in the cells, demonstrating the generation of recombinant CCHFV-mKate2. Without superinfection, no mKate2 signal was seen (Fig. 3A). To verify that CCHFV-mKate2 infection is comparable to the wild-type CCHFV, we tested a range of pharmacological inhibitors. Since CCHFV infection has been shown to depend on clathrin-dependent endocytosis, cholesterol, and low pH [13], [14], we used the following reagents: *(i)* bafilomycin A, a specific inhibitor of vacuolar-type H+-ATPase [37]; *(ii)* 5-(N-Ethyl-N-isopropyl) amiloride (EIPA), an inhibitor of the Na^+^/H^+^ exchanger that specifically blocks macropinocytosis [38]; *(iii)* nystatin, which sequesters cholesterol in the plasma membrane [39]; *(iv)* dynasore, a specific inhibitor of dynamin, which is essential for the formation of clathrin-coated and caveolae vesicles [40]; and *(v)* chlorpromazine hydrochloride (CPZ), a specific inhibitor of clathrin-dependent endocytosis [41]. Similar to wild-type virus, CCHFV-mKate2 required low pH, free cholesterol, and the formation of clathrin-coated pits to infect host cells (Fig. 3A, right panel).

MVB formation requires class III PI3K activity, which directs synthesis of the lipid phosphatidylinositol 3-phosphate (PI3P) [25], [42], [43]. The requirement for PI3K in CCHFV infection was examined using the drug LY294002, a potent and specific inhibitor of this enzyme [43], [44]. As shown in Fig. 3B, treatment of the cells with the inhibitor blocked CCHFV-mKate2 infection by approximately 80%, supporting a possible functional role for MVBs in CCHFV infection.

### Endosomal sorting complex required for transport (ESCRT) regulators control CCHFV infection

**Figure 4:**
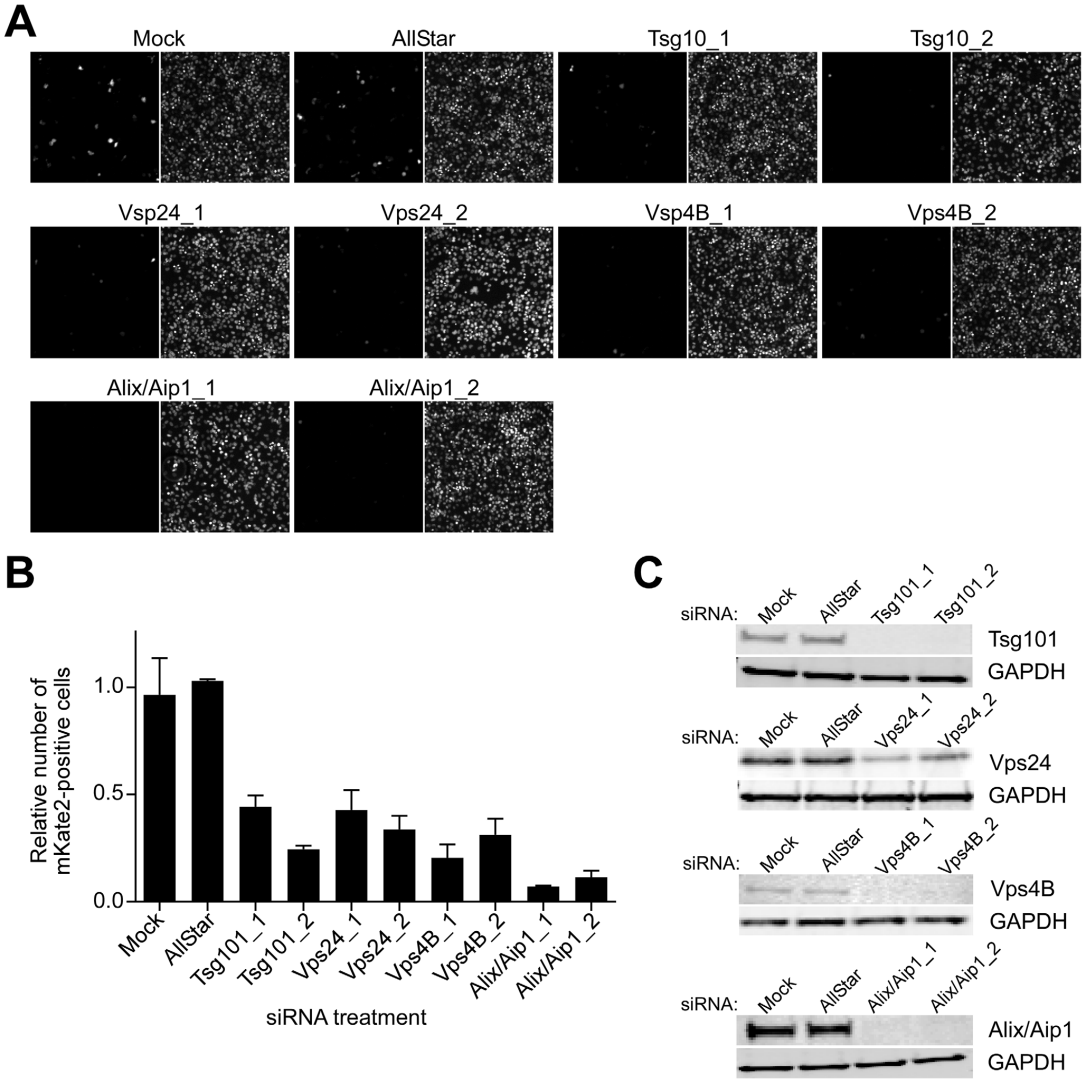
ESCRT regulators control CCHFV infection. SW13 cells were transfected with AllStar (non-targeting) siRNA, siRNAs targeting Tsg101, Vps24, Vps4B, or Alix/Aip1, or were left untreated (mock). After 24 h, the treatment was repeated. After another 24 h, the cells were split into two sets. The next day, one set of cells was challenged with CCHFV-mKate2, and lysates were collected from the second set. (A) After an additional 24 h, infected cells were fixed, treated with the Hoechst 33342 dye to stain nuclei (right panel of each image pair), photographed, and infected cells were identified by mKate2 expression (left panel of each image pair). (B) The infection efficiencies for each sample were calculated as described in Figure 3A. (C) The host protein depletion was verified by immunoblotting with anti-Tsg101, anti-Vps24, anti-Vps4B, or anti-Alix/Aip1 antibodies, and an equal number of cells in each sample was confirmed with anti-GAPDH antibody.

MVB biogenesis critically depends on a group of class E vacuolar protein sorting (Vps) regulators, which form three large hetero-oligomeric complexes within the ESCRT pathway and are designated ESCRT-I, II, and III. The cargo, which is initially selected in early endosomes, is passed on to ESCRT-I on MVB membranes, where tumor susceptibility gene 101 (Tsg101) protein plays a central role in recognizing the cargo and activating ESCRT-II and subsequently ESCRT-III complexes [45]. ESCRT-associated ATPases Vps4A and B are important in the later stages of the MVB pathway by catalyzing disassembly of ESCRT-III complex, a step critical for sorting cargo into intraluminal vesicles within MVBs [46]. Alix/Aip1 interacts with Tsg101 and components of ESCRT-III complex and, as indicated above, directly modulates the formation of intraluminal vesicles, giving rise to MVBs [31]. To further investigate whether MVB biogenesis is critical for CCHFV infection, we tested the effect of depleting cellular Tsg101, Vps24 (ESCRT-III component), Vps4B, and Alix/Aip1 (Fig. 4C) on efficiency of CCHFV-mKate2 infection. Treatment of cells with siRNAs specific to these ESCRT regulators, but not with a non-targeting siRNA, blocked infection by >60%, with Alix/Aip1 siRNAs being the most potent (Figs. 4A–B). These data demonstrate that the MVB/ESCRT pathway controls CCHFV infection.

### ESCRT regulators control CCHFV entry

**Figure 5:**
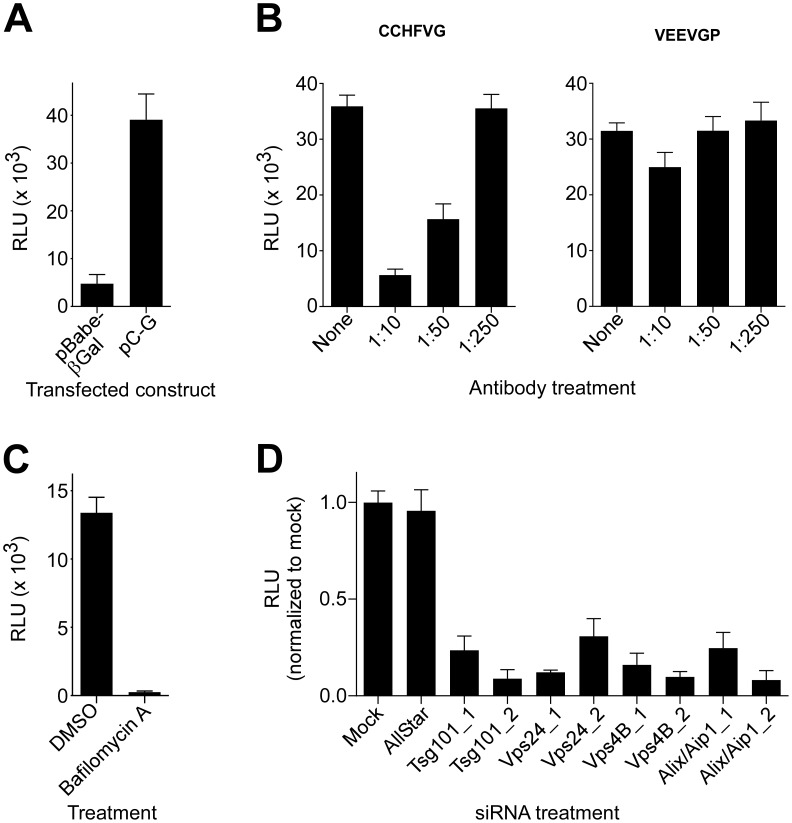
ESCRT regulators control CCHFV entry. (A) To generate VSV pseudotyped with CCHFV G (VSV-CCHFVG), 293FT cells were transfected with either pBabe-βGal (control) or pC-G. After 18 h, the cells were inoculated with a VSV-VEEVGP stock for 6 hours. The supernatants were collected 48 h after infection and incubated with SW13 cells to determine the titer of the pseudotyped virus. Luciferase activity was measured 24 h later. (B) A neutralization assay was performed by incubating VSV-CCHFVG or VSV-VEEVGP with anti-Gc antibody at the indicated dilutions for 30 min. The pseudotype-antibody mixtures were subsequently added to SW13 cells, and luciferase activity was measured 24 h later. (C) SW13 cells were preincubated with DMSO or bafilomycin A (20 nM) for 1 h and then challenged with VSV-CCHFVG in the presence of the drug. Luciferase activity was measured after 24 h. (D) SW13 cells were treated with siRNAs as described in Fig. 4. Forty-eight h later, cells were plated for assessment of host gene silencing or pseudotype infection. The following day, cells were incubated with VSV-CCHFVG or were tested for host gene silencing by immunoblotting (Figure 4C). Luciferase activity was measured 24 h after pseudotype addition to cells. The averages of three experiments and standard deviations are reported relative to mock.

Our results indicate that CCHFV is transported through MVBs and that productive virus infection requires MVB biogenesis. However, it is unclear what step of the viral lifecycle is regulated by the MVB pathway; it could be entry and/or viral gene expression and genome replication. Pseudotyped virus particles have become a valuable tool for analysis of entry mechanism of numerous viruses. However, to date, no pseudotype system has been described for CCHFV. To generate VSV pseudotyped with CCHFV glycoproteins (VSV-CCHFVG), we expressed CCHFV glycoprotein precursor G or β-galactosidase (control) in 293FT cells and then inoculated the cells with Venezuelan equine encephalitis virus glycoprotein (VEEV GP) pseudotyped VSV stock encoding a firefly luciferase reporter gene (VSV-VEEVGP). VSV-VEEVGP was used because of its ability to grow to high titers and because residual VEEV GP is short-lived in target cells (our unpublished data). As VSV core is replicated and new viral particles are assembled, the overexpressed CCHFV glycoproteins would become incorporated into nascent virions. β-galactosidase-expressing cells served as an indicator of VSV-VEEVGP carryover in the media of the inoculated cells since no viral glycoprotein was expressed there. The pseudotyped virus was collected 48 h after addition of VSV-VEEVGP and added to monolayers of SW13 cells to assess titers by measuring luciferase activity. The intensity of luminescence in cells incubated with VSV-CCHFVG was typically 8 times higher than in cells incubated with the control supernatant (Fig. 5A).

Since Gc is crucial for CCHFV binding to the cell [12], we performed a neutralization test using a polyclonal anti-Gc antibody to assess whether functional CCHFV glycoprotein complexes were present on the pseudovirion surface. VSV-CCHFVG or VSV-VEEVGP was incubated with the antibody, and the pseudotype-antibody mixtures were transferred onto SW13 cells. Luciferase activity was measured 24 h later. The antibody treatment inhibited VSV-CCHFVG, but not VSV-VEEVGP infection in a dose-dependent manner, indicating that the pseudovirus entry was glycoprotein-mediated (Fig. 5B).

CCHFV infection is pH-dependent, requiring endosomal acidification during entry ([13], [14] and Fig. 2A). To test pH dependency of VSV-CCHFVG, SW13 cells treated with DMSO or bafilomycin A were challenged with the pseudotyped virus. As seen in Fig. 5C, bafilomycin A treatment inhibited infection by >98%, demonstrating that, similarly to wild-type CCHFV, the pseudotyped virus requires acidic environment for infection and confirming that glycoprotein activation depends on low pH.

We next used VSV-CCHFVG to verify whether the MVB pathway had a role in CCHFV entry. SW13 cells depleted of cellular Tsg101, Vps24, Vps4B, or Alix/Aip1 (Fig. 4C) were incubated with VSV-CCHFVG. A recent study reporting the involvement of ESCRT regulators in arenavirus entry excluded the possibility that these ESCRT-specific siRNA treatments affected cytoplasmic transport or replication of the VSV core [25]. As seen in Fig. 5D, silencing ESCRT regulators significantly inhibited VSV-CCHFVG infection, suggesting that the MVB/ESCRT pathway is crucial for CCHFV entry.

### Lipid transport out of MVBs is dispensable for CCHFV entry

**Figure 6:**
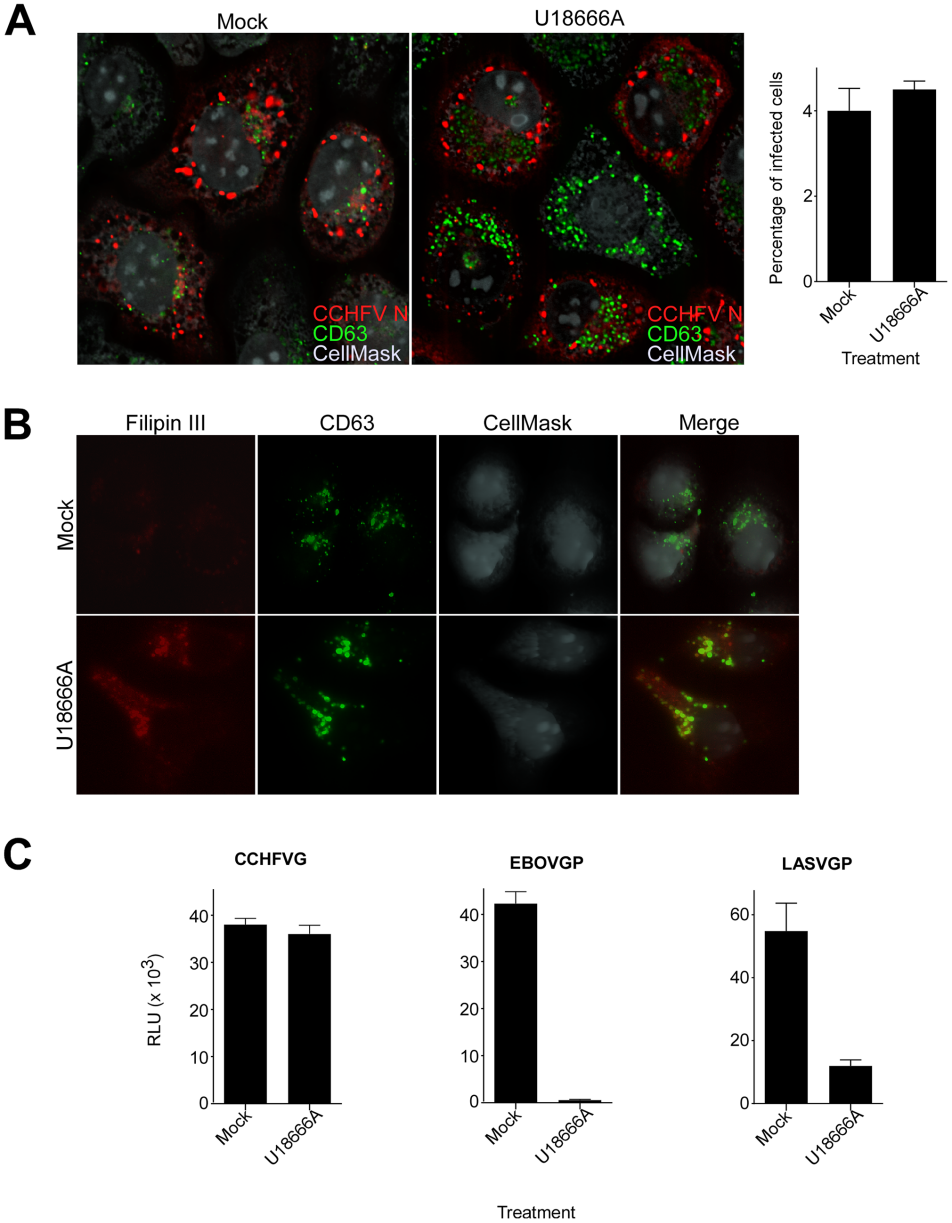
Lipid transport out of MVBs is dispensable for CCHFV entry. (A) SW13 cells were pretreated with U18666A (30 µM) for 1 h or left untreated (mock). Then, the cells were incubated with CCHFV in the presence of the drug for 24 h and subsequently fixed, permeabilized, and stained with anti-N antibody (red), anti-CD63 antibody (green), and CellMask blue dye (grey) to define cell boundaries. The samples were imaged by immunofluorescence, and an optical section through the middle of the cell is shown (left and middle panels). Relative infection efficiencies were calculated by dividing the number of infected cells by the total number of cells and are averages of three independent experiments, with error bars representing standard deviations (right panel). (B) Cells treated as described in (A) were fixed 1 h after treatment and then stained with anti-CD63 antibody (green), filipin III (red), and CellMask red dye (grey). The images were generated as described above. (C) SW13 cells were treated with U18666A (30 µM) for 1 h or left untreated (mock), then incubated with VSV-CCHFVG, VSV-EBOVGP, or VSV-LASVGP. Luciferase activity was measured 24 h after pseudotype addition.

According to our results, CCHFV is transported through MVBs and requires ESCRT regulators during entry. The drug U18666A inhibits lipid transport from MVB/late endosomal compartments [47], [48] and disrupts the trafficking of MVB-associated membrane proteins [48]–[50]. To test whether virus needs to traffic beyond MVBs, mock-treated cells or cells treated with U18666A were challenged with wild-type CCHFV for 24 h and then stained with anti-N antibody to assess infection and with anti-CD63 antibody to examine MVB morphology. We found that U18666A had no effect on virus infection as the expression level and the intracellular distribution of N or infection efficiency (approximately 4%) were similar to those in mock-treated cells (Fig. 6A). The N distribution was consistent with previously reported data [51], appearing as aggregates of various sizes close to the cell nucleus. These did not colocalize with the MVB marker CD63, despite an increase in size and number of CD63 puncta (Fig. 6A), likely due to the block of sphingolipid and cholesterol traffic out of the organelle [47]–[50]. Indeed, in agreement with previous reports [50], [52], we observed an accumulation of cholesterol in MVBs in cells treated with U18666A, but not in mock-treated cells, by staining with the cholesterol-binding compound filipin III (Fig. 6B).

We next examined whether U18666A treatment affected virus entry. Mock-treated cells or cells treated with U18666A were incubated with VSV-CCHFVG. Controls were pseudotyped viruses containing either Ebolavirus (EBOV) or LASV glycoprotein (VSV-EBOVGP and VSV-LASVGP, respectively). EBOV entry depends on the cholesterol transporter protein Niemann-Pick C1 and therefore is sensitive to U18666A treatment [53], [54]. LASV passes through MVBs during entry to fuse with late endosomal membranes where Lamp1 serves as a fusion receptor [25], [55]. We found that the inhibitor had no effect on VSV-CCHFVG infection, while infections of VSV-EBOVGP and VSV-LASVGP were blocked by >98% and >85%, respectively (Fig. 6C), suggesting that lipid traffic out of MVBs is not required for CCHFV entry. These data and findings that Rab7 does not play a role in CCHFV infection [13] or localization to MVBs (Fig. 2C) demonstrate that virus does not require trafficking beyond MVBs during entry.

### Bafilomycin A treatment results in accumulation of CCHFV in MVBs

**Figure 7:**
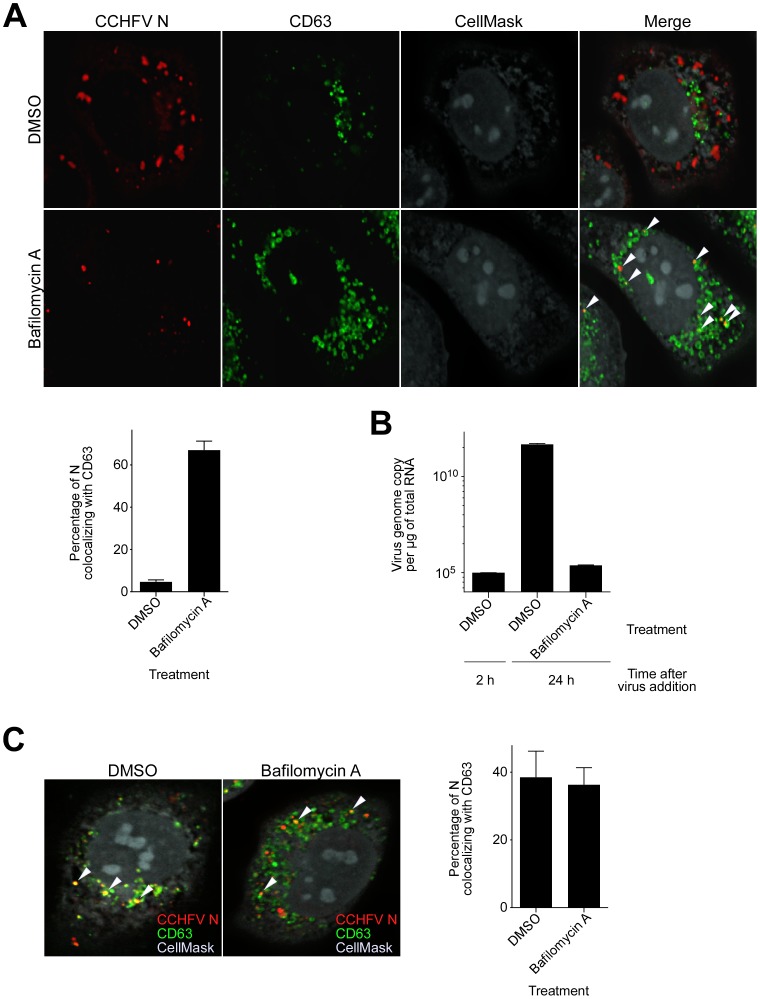
Bafilomycin A treatment results in accumulation of CCHFV in MVBs and blocks virus replication. (A) SW13 cells were pretreated with DMSO or bafilomycin A (20 nM) for 1 h. Subsequently, cells were incubated with CCHFV in the presence of the drug for 24 h and then fixed, permeabilized, and stained with anti-N antibody (red), anti-CD63 antibody (green), and CellMask blue dye (grey). Images were obtained as Z-stacks, and 3D images of cells were generated to assess colocalization between N and MVBs. An optical section through the middle of the cell is shown (top and middle panels). The arrowheads indicate examples of CCHFV N-CD63 colocalization (yellow). The percentages of N puncta found in MVBs were counted in 20 cells in each sample, and averages and standard deviations are shown (lower left panel). (B) To determine whether bafilomycin A treatment affected CCHFV replication, SW13 cells were pretreated with DMSO in duplicate or bafilomycin A for 1 h and then inoculated with equal amount of CCHFV in the presence of the drug. After 2 h, one set of DMSO-treated cells was subjected to RNA extraction. RNA isolation from the second set of DMSO-treated cells and bafilomycin A-treated cells took place 24 h after virus addition. Viral RNA levels were determined by a qRT-PCR (TaqMan) assay detecting sequences in S segment of the genome. Two independent experiments were performed in duplicate, and standard deviations were calculated. Representative data are shown. (C) SW13 cells were treated as described in (A), then incubated with gamma-irradiated CCHFV in the presence of the drug for 24 h. Antibody staining and quantification of N-CD63 colocalization were performed as in (A) (right panel).

Our results indicate that MVBs might represent the last endocytic compartment before CCHFV escape into the cytoplasm for replication (Figs. 2C and 6). In Fig. 3A and work by others [13], [14], the virus also requires an acidic environment to establish productive infection. Therefore, blocking endosomal acidification should trap virus in vesicles prior to sites of membrane fusion. Cells treated with bafilomycin A or DMSO were incubated with wild-type virus for 24 h. We then assessed the intracellular distribution of viral N protein and N-MVB colocalization. According to both Simon *et al.* [14] and our findings (Fig. 5C), inhibition of vesicular acidification for 24 h blocked virus entry. We observed that, while DMSO treatment did not affect virus infection, treatment with bafilomycin A resulted in accumulation of N, presumably virions, within MVBs (Fig. 7A, top and middle panels). The amounts of N found in MVBs were 4% in DMS0-treated cells and 67% in bafilomycin A-treated cells (Fig. 7A, lower left panel).

Next, we determined whether virus genome replication took place in MVBs. Cells treated with DMSO in duplicate or bafilomycin A were inoculated with CCHFV. We then isolated RNA from one set of DMSO-treated cells 2 h later and from the other two samples 24 h after virus addition. The RNA was used to determine virus genome copies present, where the sample collected from the first set of DMSO-treated cells served as a control, since virus genome replication is not detectable 2 h after incubating cells with virus [14]. As seen in Fig. 7B, no significant difference in the genome copy number was observed between the control sample and the bafilomycin A-treated sample. In contrast, the second set of DMSO-treated cells exhibited a 6-log increase in genome equivalents, demonstrating that bafilomycin A treatment blocks CCHFV replication. To test whether virus replication was a requirement for virus localization to MVBs, we repeated the experiment identical to that described in Fig. 7A but used CCHFV that was inactivated by gamma-irradiation. We observed that approximately 40% of virions associated with MVBs in cells that were treated with either DMSO or bafilomycin A (Fig. 7C), demonstrating that CCHFV association with MVBs is independent of genome replication. Altogether, our findings suggest that CCHFV membrane fusion or capsid penetration into the cell cytoplasm likely occurs from MVB compartments.

## Discussion

Entry into host cells is the first committal step in any virus replication cycle. In this study, we characterized the entry route taken by CCHFV after internalization from the plasma membrane. We confirmed previous work that the virus was transported through early endosomes in a Rab5A-dependent manner, but additionally showed that MVBs play an important role in productive infection. Virions localized to and reorganized the intracellular distribution of MVBs, and ESCRT pathway-related proteins involved in MVB formation and function were needed for entry. We also determined that endocytic trafficking out of MVBs did not affect CCHFV infection and that blocking endosome acidification resulted in accumulation of virions in the MVBs. These findings indicate that during entry the MVB is a late stage destination of CCHFV particles and is likely the site from which they are released into the cell cytoplasm to initiate genome replication.

CCHFV enters cells by clathrin-dependent endocytosis [13], [14]. The process is rapid, with endocytosed ligands and receptors being sorted to different organelles for processing [56]–[59]. Early endosomes have been considered to be initial sorting organelles [57], [59], and since CCHFV infection was shown to require Rab5, the virus was thought to traffic through this compartment [13]. However, actual localization of virions to early endosomes was not previously demonstrated. In the current study, we used microscopy to track movement of CCHF virions through endocytic compartments. Virions were found in early endosomes starting at 15 min post-infection (Fig. 1A), although we do not exclude the possibility that the virus localized to early endosomes at even earlier time points, as has been demonstrated for influenza A virus [59]. The increase in percentage of viral particles in the endosomes at later time points, however, indicates that CCHFV may enter host cells at a slower pace, reflecting potential differences in endosomal kinetics in different cell lines, markers used to identify the endosomes, or viral entry mechanisms. Recently, Lakadamyali *et al.* suggested that early endosomes consist of two populations with distinct maturation kinetics, designated dynamic and static [59]. Cargo in the static population undergoes endocytosis less rapidly and depends, at least in part, on the presence of adaptor protein complex 2 (AP-2) [59], [60]. CCHFV infection requires AP-2 [13], while influenza A virus infection does not [59], suggesting that these two viruses may utilize different sets of early endosomes for uptake into cells.

Cargo sorted in early endosomes can be progressively shuttled along the endocytic pathway toward MVBs [23], [24]. Several viruses, including those entering cells by clathrin-mediated endocytosis, have been demonstrated to localize to MVBs and depend on the sorting protein complex, ESCRT, during infection. Influenza A virus, LASV, and LCMV appear to pass through MVBs on their way toward late endosomes, while VSV needs the compartment to release genome into the cytoplasm for replication [25]–[28]. For CCHFV, MVB involvement seems to be distinct. We found that CCHF virions localized to and dramatically affected intracellular distribution of MVBs (Fig. 2A). ESCRT regulators controlled CCHFV entry (Figs. 4 and 5D), and trafficking beyond MVBs appeared to be dispensable for virus infection and entry (Figs. 2C and 6), suggesting that MVBs might be the last endosomal compartment before virus exit into the cytoplasm. The small number of virus particles in Lamp1-positive compartments (Fig. 2B) raised a question about a role, if any, of late endosomes or lysosomes in CCHFV infection. Since Rab7 controls vesicular transport out of MVBs [32] and is not essential for virus infection [13], it is unlikely that localization of virus particles to compartments downstream of MVBs has functional significance. Additionally, a small amount of Lamp1 is found in MVBs [61]–[63], so CCHF virions might have localized to these Lamp1-positive MVBs. We detected virus particles by staining infected cells with an anti-N antibody, and therefore our data indicate that MVBs may be where virus-endosome fusion takes place or where, similarly to VSV, nucleocapsids are released into the cytoplasm, or both, provided membrane fusion and nucleocapsid release are concurrent. Because our data also showed that blocking vesicular acidification resulted in inhibition of a novel CCHFV pseudotype (Fig. 5C), affecting glycoprotein function and therefore virus entry, and accumulation of N, presumably virions, in MVBs (Figs. 7A and 7C), MVBs are likely the site of fusion of the viral and host membranes. The role of the MVB redistribution during CCHFV infection is unclear and requires additional examination, but, to our knowledge, was not observed during infection by any other virus.

While we identified Rab5A as a factor controlling CCHFV traffic from the plasma membrane to early endosomes (Fig. 1B), it is less clear how virions are transported from early endosomes to MVBs. Rab7 is known to localize on a variety of organelles including early and late endosomes, MVBs, and lysosomes [32], [64], [65]. It is unclear, however, whether Rab7 regulates transport between these organelles or is itself trafficked via each to reach compartment(s) it functions on. A study by Vanlandingham and Ceresa showed that silencing Rab7 expression had no effect on MVB biogenesis, but instead affected lysosomal degradation of the epidermal growth factor in complex with its receptor by blocking transport/fusion between MVBs and lysosomes [32]. Our current observations (Fig. 2C) are in agreement with those findings, demonstrating that for CCHFV, Rab7A does not contribute to early endosome-MVB transport and therefore does not control CCHFV infection, which is consistent with previously reported data [13]. Additional factors controlling the traffic between early endosomes and MVBs remain to be identified.

The formation of internal vesicles within MVB compartments requires class III PI3K, whose cellular role is to facilitate synthesis of PI3P lipid [25], [42], [43]. Protein sorting machinery, which selects cargo in early endosomes, is thought to directly bind early endosome-specific PI3P [66], [67] and subsequently recruit ESCRT complexes to MVB membranes [68]. Inhibition of PI3K activity, therefore, results in formation of defective MVB compartments devoid of internal vesicles and thus missorted cargo. In the presence of PI3K inhibitor LY294002, CCHFV infection was significantly reduced (Fig. 3B), most likely due to the block in MVB biogenesis. In addition to its role in vesicle formation and membrane trafficking, PI3K pathway is known to regulate various cellular functions such as survival and proliferation, glucose transport, superoxide production, and motility through actin rearrangement [69]. There is mounting evidence that the PI3K pathway is often disregulated in human tumors [70]. To date, several promising PI3K inhibitors have entered human trials for cancer treatments [71], [72]. Our current results that the PI3K pathway is also required for CCHFV infection could be exploited for development of new therapeutics to treat the CCHFV disease.

One of the limiting factors in studying early steps in CCHFV infection has been the lack of a pseudotyped virus. Although pseudotype systems have been described for several bunyaviruses [73]–[76], no such system was reported for CCHFV to date. Here, we reported a VSV core virus pseudotyped with CCHFV glycoprotein. Treatment of the pseudotyped virus with anti-Gc antibody or bafilomycin A (Figs. 5B–C) inhibited its infection, indicating that the entry was glycoprotein-dependent. The finding that neutralizing antibody blocked pseudotype infection also demonstrated that the pseudotype can be used in rapid diagnostic assays, circumventing the need for high-level biosafety containment and pathogenic virus stocks.

The bunyavirus family consists of five genera with over 350 members [5]. Of those tested, each require low pH to productively infect host cells and so, require uptake into acidified endosomes [22], [77]–[81]. CCHFV belongs to the *Nairovirus* genus, and its entry route is similar to that used by members of the *Orthobunyavirus* genus, particularly La Crosse and Oropouche viruses. La Crosse virus infection was shown to be inhibited by overexpression of DN Rab5, but not DN Rab7, leading to the conclusion that this virus undergoes fusion at early endosomes [77], as was thought to occur for CCHFV [32]. While this might be the case, given our findings, MVBs may play a similar role in La Crosse virus infection (Figs. 2A–B). For Oropouche virus, virions were not seen associated with early endosomes, but did localize to Rab7-positive endosomes [80], indicating that the virus may bypass the early endosome compartment. However, the location of Oropouche virus particles in cells was only examined after 40 minutes. This seemingly different mechanism of entry may be explained by the rapid and/or transient movement of virions through early endosomes, which, for CCHFV, occurred as early as 15 minutes after incubation with cells. The finding that Oropouche virus was Rab7-associated does suggest trafficking to late endosomes; however, Rab7 is not ideal for identifying the exact endosomal compartment since it localizes to various organelles, including subsets of MVBs, late endosomes, and lysosomes [32]. It is, therefore, important to determine if either La Crosse or Oropouche virus requires the MVB for infection or if CCHFV has a unique need for this compartment. In contrast, Hantaan and UUkuniemi viruses, each belonging to the *Hantavirus* and *Phlebovirus* genera, do require trafficking through and function of late endosomes or lysosomes [22], [79]. For Hantaan virus, virions were associated with Lamp1-positive endosomes, although functional significance of this association was not investigated [79]. A comprehensive study by Lozach *et al.* used a combination of microscopy as well as overexpression of DN and CA forms of Rab5 and Rab7 to track UUkuniemi virions through the endosomal network. Their findings convincingly demonstrate that UUkuniemi virus infection depends on Rab7 and requires virion localization to late endosomes or lysosomes [22]. Unfortunately, passage of virus particles through MVBs was not studied. These differences in entry mechanisms for viruses may reflect the broad diversity of viruses across the *Bunyaviridae* family.

In conclusion, our work has identified a novel requirement for trafficking of CCHFV through MVBs and has shown that host proteins controlling MVB function are important host factors needed for CCHFV infection and entry. Our findings were substantiated through the production and use of a novel pseudotyped virus bearing the glycoprotein of CCHFV. This reagent will most certainly stimulate further studies on entry mechanism of the virus as well as serve as a safe diagnostic tool overcoming the need to cultivate wild-type, pathogenic virus. The knowledge acquired during the studies will help to design new strategies for therapeutic intervention against this pathogen.

## Materials and Methods

### Cells

Human adrenal gland carcinoma (SW13; ATCC #CCL-105) cells were cultivated in Dulbecco's modified Eagle's medium (DMEM) supplemented with 10% fetal bovine serum (FBS). 293FT (Life Technologies, Carlsbad, CA) and baby kidney hamster-derived BsrT7/5 cells [36] were cultivated in DMEM supplemented with 10% FBS and 0.5 mg/mL G418 (Life Technologies, Carlsbad, CA). All cells were maintained at 37°C with 5% CO_2_.

### Antibodies and inhibitors

Rabbit antibody to CCHFV N was generously provided by Ali Mirazimi (Karolinska Institutet, Sweden), and mouse antibody to CCHFV N was provided by Connie Schmaljohn (U.S. Army Medical Research Institute of Infectious Diseases, Washington, D.C.). The mouse antibody to CD63, clone H5C6, developed by J. Thomas August and James E.K. Hildreth (Johns Hopkins University School of Medicine) was obtained from the Developmental Studies Hybridoma Bank, created by the National Institute of Child Health and Human Development (NICHD), and maintained at The University of Iowa, Department of Biology, Iowa City. Mouse antibodies to Tsg101 and rabbit antibodies to Vps24, Vps4B, and PDCD6 (referred to as Alix/Aip1) were from Abcam, Cambridge, MA. Other antibodies used here were mouse antibody to GAPDH (Life Technologies, Carlsbad, CA), mouse antibody to EEA1 (BD Biosciences, Franklin Lakes, NJ), mouse antibody to Lamp1 (Santa Cruz Biotechnologies, Dallas, TX), and rabbit antibody to CCHFV Gc (IBT Bioservices, Gaithersburg, MD).

Dimethylsufoxide (DMSO) was from ATCC (Manassas, VA); 5-(N-Ethyl-N-isopropyl) amiloride (EIPA) was from Sigma (St. Louis, MO); LY294002, U18666A, bafilomycin A, dynasore, nystatin, and chlorpromazine hydrochloride (CPZ) were from EMD Millipore (Billerica, MA).

### Viruses

All experiments with infectious CCHFV strain IbAr10200 were performed in the biosafety level 4 (BSL-4) laboratory at the Texas Biomedical Research Institute (San Antonio, TX). The virus was amplified in SW13 cells in DMEM containing 2% FBS for 5 days. To determine the titer of the virus stock, SW13 cells were incubated with 10-fold serial dilutions of the virus at 37°C for 1 h. After the virus was removed, the cells were overlaid with DMEM containing 2% FBS and 1.5% methyl cellulose (Sigma, St. Louis, MO). Forty-eight hours later, cells were fixed in 10%-buffered formalin (Sigma, St. Louis, MO) for 24 hours. Then, the cells were stained with the anti-N antibody, and fluorescent foci were counted to determine the virus titer.

To generate a CCHFV stock for gamma-irradiation, supernatants of SW13 cells infected with the virus for 5 days were clarified of debris, laid over a cushion of 20% sucrose in PBS, and centrifuged for 3 h at 25,000 rpm at 4°C. The pellet containing concentrated virus was resuspended in PBS and stored at −80°C. The sample was subjected to a single dose of 5 Mrad cobalt-60 gamma-irradiation at the BSL-4 laboratory at the University of Texas Medical Branch, Galveston, TX. The inactivation of the virus was confirmed by a plaque assay.

Recombinant vesicular stomatitis virus (VSV), containing a substitution of VSV glycoprotein G gene with a gene encoding firefly luciferase was generously provided by Sean Whelan (Harvard Medical School, Boston, MA).

### Plasmids

The codon-optimized cDNA of CCHFV G and N (strain IbAr10200), and L (strain Sudan) were synthesized and cloned into pcDNA3.1(+) vector (Life Technologies, Carlsbad, CA) by Epoch Life Science (Missouri City, TX), producing pcDNA-G, pcDNA-N, and pcDNA-L, respectively. The G gene was subsequently amplified by PCR with a forward primer (5′-ttttggcaaa GAATTC ATG CAT ATC AGC CTC ATG TAC GCT ATC TTG-3′) and a reverse primer (5′- cgggggtaccCTCGAG CTA GCC TAT ATG TGT TTT TGT GCT AAA CAG CTC-3′). The PCR product was cloned into the protein expression vector pCAGGS/MCS [82], [83] using In-Fusion cloning technology (Clontech, Mountain View, CA) to generate pC-G.

To obtain pT7-mKate2, the cDNA of the red fluorescent protein mKate2 in the antisense orientation between the 5′ and 3′ terminal non-coding regions (NCRs) of the CCHFV M segment and flanked by the T7 RNA polymerase promoter and a ribozyme was synthesized by Epoch Life Science. The cDNA digested with ClaI and SalI was then subcloned into a vector backbone generated by excising the Ebolavirus minigenome from the p3E5E-Luc [84] (a generous gift of Elke Muhlberger (Boston University)). To obtain pT7-Sseg, two-step PCR was performed. In the first step, culture supernatant of SW13 cells infected with CCHFV was used as a template for reverse transcription (RT)-PCR to amplify S segment with a forward (5′-TCT CAA AGA AAC ACG TGC CGC TTA C-3′) and a reverse (5′-TCT CAA AGA TAC CGT TGC CGC AC-3′) primers. A ribozyme cDNA was generated by PCR using pT7-mKate2 as a template and a forward (5′-GTA AGC GGC ACG TGT TTC TTT GAG A GGG TCG GCA TGG CAT CTC CAC-3′) and a reverse (5′-ACG TCC TCC TTC GGA TGC CC-3′) primers. In the second step, the S segment and ribozyme PCR products served as templates for a PCR reaction to obtain cDNA containing S segment sequence in negative orientation flanked by the T7 RNA polymerase promoter and a ribozyme. The primers were a forward primer containing a sequence of a T7 promoter (5′-TGC AGG GGG AT ATCG AT Ta ata cga ctc act ata G TCT CAA AGA TAC CGT TGC CGC AC-3′) and a reverse primer (5′-ATG CCT GCA GGT CGA C ACG TCC TCC TTC GGA TGC CC-3′). The cDNA was cloned into a backbone generated by excising the Ebolavirus minigenome from the p3E5E-Luc between ClaI and SalI restriction sites. Cloning was performed using In-Fusion cloning technology. Four different clones were subjected to DNA sequencing to establish an S segment consensus sequence and to verify successful fusion of the virus segment to the T7 promoter and ribozyme. The consensus sequence was determined to be identical to the one with NCBI accession number NC_005302.

pBabe-βGal and pLenti-eGFP plasmids were described previously [85], [86].

The dominant negative forms (DN) of Rab5A and Rab7A containing S34N and T22N substitutions [18], [35], respectively, were cloned into pLenti-eGFP plasmid as fusions to the C-terminus of eGFP, yielding pRab5A-DN and pRab7A-DN. The cloning was performed by Epoch Life Science, Houston, TX. The constitutively active form (CA) or Rab5A containing Q79L substitution [19] was reported previously [87].

### siRNA

siRNA duplexes targeting human HRS, Tsg101, Vps22, Vps24, Vps4B, and Alix/AIP1 genes and AllStar non-silencing siRNA were purchased from Qiagen (Germantown, MD). The siRNA sequences are available upon request.

SW13 cells grown in 12-well plates were transfected with siRNA duplexes to a final concentration of 5 nM using RNAiMAX transfection reagent (Life Technologies, Carlsbad, CA) according to the manufacturer's protocol. Twenty-four h later, the transfection was repeated. Cells were incubated with the transfection mixtures for another 24 h.

### Immunoblotting

siRNA-transfected cells were lysed in RIPA buffer (50 mM Tris-Cl [pH 7.5], 150 mM NaCl, 1% Triton X-100, and 0.1% SDS). The lysates were kept on ice for 15 minutes and then clarified of debris by centrifugation. The lysates were incubated with SDS-PAGE sample buffer at 100°C for 10 minutes, and proteins were resolved on a 4%–20% SDS-PAGE gradient gel (Bio-Rad Laboratories, Hercules, CA). The samples were transferred onto iBlot nitrocellulose membranes (Life Technologies, Carlsbad, CA) and blocked for 1 h at room temperature with a blocking buffer (LI-COR Biosciences, Lincoln, NE). The blots were incubated with primary antibodies at 4°C overnight and then with anti-rabbit IRDye 800CW and anti-mouse IRDye 680LT (LI-COR Biosciences, Lincoln, NE) for 1 h at room temperature. Protein bands were visualized using Odyssey software (LI-COR Biosciences, Lincoln, NE).

### Generation of CCHFV-mKate2

BsrT7/5 cells were transfected with 0.25 µg of each pT7-mKate2, pcDNA-N, and pcDNA-L using the Neon transfection system (Life Technologies, Carlsbad, CA) according to the manufacturer's protocol. The electroporated cells were plated into wells of 12-well dishes. After 24 h, cells were either infected with CCHFV strain IbAr10200 at multiplicity of infection (MOI) of 0.1 or left uninfected. Forty-eight h after infection, supernatants were added to SW13 cells at dilution of 1∶10 to assess the minigenome packaging. The SW13 cells were fixed in 10% formalin 24 h after addition of the recombinant virus. Cells nuclei were stained with Hoechst 33342 dye, and analyzed as described above. The number of mKate2-expressing cells infected with the recombinant virus was approximately 4%.

### Pseudotyped VSV

To generate CCHFV G pseudotyped VSV encoding the firefly luciferase gene (VSV-CCHFVG), 293FT cells grown in 100-mm dishes were transfected with either 15 µg of pBabe-βGal (control) or 5 µg of pC-G and 10 µg of pBabe-βGal using CaCl_2_ method. After 18 h, media was replaced, and the cells were inoculated with 1 ml of a previously prepared Venezuelan equine encephalitis virus glycoprotein pseudotyped VSV stock (VSV-VEEVGP). After another 6 hours, the supernatants containing the virus inoculum were replaced with fresh media. The pseudotyped virus was collected 48 h after infection. Virus titers were determined in SW13 cells grown in 96-well plates using 5 times dilution. Twenty-four hours later, luciferase activity was measured using Steady-Glo luciferase assay buffer (Promega, Madison, WI) according to the supplier's protocol and a GloMax-96 microplate luminometer (Promega, Madison, WI). Neutralization tests were performed by incubating VSV-CCHFVG or VSV-VEEVGP with a rabbit anti-Gc antibody to the antibody dilution of 1∶10, 1∶50, or 1∶250. After incubation at room temperature for 30 min, the pseudotype-antibody mixtures were transferred onto SW13 cells grown in a 96-well plate. The luciferase activity was assessed 24 h later as described above. To test pH dependency of VSV-CCHFVG, SW13 cells grown in a 96-well plate were incubated with DMSO or bafilomycin A dissolved in DMSO to a final concentration of 20 nM. One h later, cells were infected with VSV-CCHFVG, and luciferase activity was measured 24 h later.

Generation of VSV pseudotyped with either Ebolavirus glycoprotein GP or Lassa virus glycoprotein GP and encoding the firefly luciferase gene (VSV-EBOVGP and VSV-LASVGP, respectively) was described previously [88]–[90].

### Virus infection

To test the effect of gene silencing on CCHFV infection, siRNA-treated SW13 cells were collected 24 h after second siRNA transfection. Fifteen thousand cells were plated into wells of the 96-well plate in triplicate to be infected with CCHFV-mKate2, and the remaining cells were replated to be tested for gene silencing. Twenty-four hours later, virus was added to the cells at 10 times dilution, and the gene expression was verified by immunoblotting as described above. Infected cells were fixed in 10% buffered formalin 24 h later, for 24 h. Cell nuclei were stained with Hoechst 33342 dye (Life Technologies, Carlsbad, CA). Cells were photographed using Nikon Ti-Eclipse microscope running high content analysis software (Nikon, Tokyo, Japan). The numbers of cell nuclei and mKate2-expressing (infected) cells were counted using CellProfiler software (Broad Institute, Boston, MA) with parameters developed in our laboratory (available upon request). The infection rate was calculated as the ratio of infected cells to cell nuclei.

To assess the effect of pharmacological treatment on CCHFV-mKate2 infection, SW13 cells were plated into a 96-well plate at 15,000 cells per well. After 24 h, cells were incubated with one of the following: bafilomycin A (to the final concentration of 10 nM), EIPA (10 µM), dynasore (200 µM), nystatin (100 µM), CPZ (10 µg/mL), or LY294002 (75 µM). The control was DMSO treatment. All treatments were performed in triplicate. One h later, cells were infected with CCHFV-mKate2 as described above. Cells were fixed with 10% formalin, stained with Hoechst 33342 dye, and analyzed as above.

To test the effect of U18666A on virus infection, SW13 cells grown in 8-chamber μ-slides (ibidi, Munich, Germany) were treated in duplicate with U18666A to the final concentration of 30 µM or with H_2_O (control). One hour later, one set of cells was infected with CCHFV for 24 h and then fixed in 10% formalin, permeabilized with 0.1% Triton X-100 in PBS for 10 minutes, blocked with 5% goat serum (Life Technologies, Carlsbad, CA) in PBS, and stained with CellMask blue dye to define cell boundaries (Life Technologies, Carlsbad, CA), rabbit anti-N antibody to identify infected cells and mouse anti-CD63 antibody at 4°C overnight. The secondary antibodies were Alexa Fluor-conjugated anti-rabbit and anti-mouse antibodies (Life Technologies, Carlsbad, CA). The block of cholesterol transport out of the MVBs in the U18666A-treated cells was confirmed by staining the second set of cells with anti-CD63 antibody followed by an Alexa Flour-conjugated secondary antibody, filipin III (Thermo Fisher Scientific, Waltham, MA), and CellMask red dye (Life Technologies, Carlsbad, CA). Z-stack immunofluorescence imaging was done on 20 cells in each sample. 3D image reconstruction was performed using Imaris software (Bitplane, Zurich, Switzerland) after image deconvolution by AutoQuant X3 software (MediaCybernetics, Rockville, MD). The experiment was repeated three times. Numbers of cells and N-positive cells were counted using CellProfiler software. The relative infection efficiencies were calculated by dividing the number of infected cells by the number of the cells in that sample.

To test the effect of CA Rab5A on virus infection, SW13 cells transfected with 0.1 µg of either pLenti-eGFP or the CA by electroporation were plated into 8-chamber μ-slides. Twenty-four h later, cells were incubated with CCHFV for 24 h, then fixed and stained with the HCS CellMask blue stain to define cell boundaries and a rabbit antibody to N to detect infected cells. Twenty eGFP-expressing cells in each sample were used to determine infection efficiency. The number of cells expressing both eGFP and N in each sample was divided over the total number of eGFP-positive cells in the sample to determine infection efficiency. The experiment was repeated three times.

### Virus internalization

All immunofluorescence experiments were repeated three times. To test the colocalization between CCHFV and early endosomes, MVBs, or late endosomes/lysosomes, SW13 cells grown in 8-chamber μ-slides were incubated with the virus for indicated times at 37°C. Then, the samples were fixed, permeabilized, and stained with antibodies: *(i)* a rabbit antibody to N and a mouse antibody to EEA1, *(ii)* a rabbit antibody to N and a mouse antibody to CD63, *(iii)* a mouse antibody to N and a rabbit antibody to PDCD6 (detecting Alix/Aip1), or *(iv)* a rabbit antibody to N and a mouse antibody to Lamp1 at 4°C overnight. The secondary antibodies were Alexa Fluor-conjugated anti-rabbit and anti-mouse antibodies. Then, the cells were stained with the HCS CellMask blue stain to identify cells. The Z-stack immunofluorescence imaging, 3D reconstruction, and deconvolution were done on 20 cells in each sample as described above. The virion-endosome colocalization was computed using Imaris software.

To assess whether the overexpression of the DN Rab5A or DN Rab7A affected the localization of virions to endosomes, SW13 cells transfected with 0.1 µg of pLenti-eGFP or a DN by electroporation were plated into wells of 8-chamber slides. After 24 h, cells were incubated with CCHFV for indicated time points. Subsequently, cells were fixed, stained, and analyzed as described above.

To test the effect of bafilomycin A on virus internalization, SW13 cells grown in 8-chamber μ-slides were treated with bafilomycin A to a final concentration of 20 nM or with DMSO as a control. One hour later, cells were incubated with either infectious or gamma-irradiated CCHFV. After 24 h, samples were fixed in 10% formalin, permeabilized, and stained with CellMask blue dye to define cell boundaries, anti-N and anti-CD63 antibodies. Immunofluorescence imaging and 3D reconstruction were done on 20 cells in each sample as described above. The percentages of N found in MVBs were computed by Imaris software.

### Real-time quantitative (q)RT-PCR

To determine whether bafilomycin A treatment affected CCHFV replication, SW13 cells grown in three 35-mm dishes were treated with DMSO in duplicate or bafilomycin A to a final concentration of 20 nM. One h later, all dishes were inoculated with an equal amount of CCHFV. After 2 h, one set of DMSO-treated cells was subjected to RNA extraction using TRIzol reagent (Life Technologies, Carlsbad, CA) according to manufacturer's protocol. RNA isolation from the second set of DMSO-treated cells and bafilomycin A-treated cells took place 24 h after virus addition. Five hundred ng of extracted RNA was used to calculate CCHFV genome copies in the samples. Viral RNA levels were determined by a qRT-PCR (TaqMan) assay detecting sequences in the S segment of the genome using an ABI 7500 real-time PCR system (Applied Biosystems, Foster City, CA). To obtain a standard curve to determine CCHFV genome equivalents, synthetic CCHFV S segment RNA was generated. pT7-Sseg was linearized with SalI (New England Biolabs, Ipswich, MA) and used as a template to synthesize RNA using MEGAscript T7 kit (Life Technologies, Carlsbad, CA) according to the manufacturer's protocol. The RNA was purified using RNAzol-Bee reagent (Tel-Test, Friendswood, TX) and resuspended in diethyl pyrocarbonate-treated water (Life Technologies, Carlsbad, CA) to 2×10^5^ copies per µl. Then, serial 10-fold dilutions of the stock were used to create a linear regression of the standard curve. The CCHFV primer/probe sequences span from nucleotides 1086 to 1216 and were a forward primer (5′-CTT TGC CGA TGA TTC TTT CC-3′), a reverse primer (5′-GAC TTA GTG TGT CCA GAT CC-3′), and a FAM/TAMRA-labeled primer (5′-TTG GGC AGC ATC ATC AGG ATT GGC-3′). The experiment was repeated twice in duplicate.
